# Altered Dark- and Photoconversion of Phytochrome B Mediate Extreme Light Sensitivity and Loss of Photoreversibility of the phyB-401 Mutant

**DOI:** 10.1371/journal.pone.0027250

**Published:** 2011-11-03

**Authors:** Éva Ádám, Andrea Hussong, János Bindics, Florian Wüst, András Viczián, Marcus Essing, Mátyás Medzihradszky, Stefan Kircher, Eberhard Schäfer, Ferenc Nagy

**Affiliations:** 1 Institute of Plant Biology, Biological Research Centre, Szeged, Hungary; 2 Institut für Biologie II/Botanik, Universität Freiburg, Freiburg, Germany; 3 Centre of Biological Signalling (BIOSS), Universität Freiburg, Freiburg, Germany; 4 School of Biological Sciences, University of Edinburgh, Edinburgh, United Kingdom; USDA-ARS, United States of America

## Abstract

The *phyB-401* mutant is 10^3^ fold more sensitive to red light than its wild-type analogue and shows loss of photoreversibility of hypocotyl growth inhibition. The *phyB-401* photoreceptor displays normal spectral properties and shows almost no dark reversion when expressed in yeast cells. To gain insight into the molecular mechanism underlying this complex phenotype, we generated transgenic lines expressing the mutant and wild-type phyB in *phyB-9* background. Analysis of these transgenic lines demonstrated that the mutant photoreceptor displays a reduced rate of dark-reversion but normal P_fr_ to P_r_ photoconversion *in vivo* and shows an altered pattern of association/dissociation with nuclear bodies compared to wild-type phyB. In addition we show (i) an enhanced responsiveness to far-red light for hypocotyl growth inhibition and *CAB2* expression and (ii) that far-red light mediated photoreversibility of red light induced responses, including inhibition of hypocotyl growth, formation of nuclear bodies and induction of *CAB2* expression is reduced in these transgenic lines. We hypothesize that the incomplete photoreversibility of signalling is due to the fact that far-red light induced photoconversion of the chromophore is at least partially uncoupled from the P_fr_ to P_r_ conformation change of the protein. It follows that the *phyB-401* photoreceptor retains a P_fr_-like structure (P_r_^*^) for a few hours after the far-red light treatment. The greatly reduced rate of dark reversion and the formation of a biologically active P_r_^*^ conformer satisfactorily explain the complex phenotype of the *phyB-401* mutant and suggest that amino acid residues surrounding the position 564 G play an important role in fine-tuning phyB signalling.

## Introduction

Plants are sessile organisms, which have established a considerable plasticity of development to respond to changes in the natural environment. Light, a highly variable environmental factor is used not only as the main energy source, but also as an environmental cue to regulate plant growth and development. To monitor these changes, plants have evolved several classes of photoreceptors: the UV-B photoreceptors [1], [2], the blue/UV-A sensing cryptochromes controlling plant development [3], the phototropins controlling directional growth, chloroplast re-orientation and stomata opening [4], [5] and the red/far-red absorbing phytochromes controlling plant development [6], [7], [8].

In *Arabidopsis thaliana* phytochromes are encoded by a small gene family of five members, *PHYA* to *PHYE* [9]. The phytochromes i*n vivo* exist as homo and/or heterodimers of about 125 kDa subunits [10] and each subunit contains a covalently linked, linear tetrapyrrole chromophore. The phytochromes are synthesized in their R light absorbing, biologically inactive P_r_ form in darkness. Absorption of R light converts them into their FR light absorbing biologically active P_fr_ form that again can be converted back into P_r_ form upon absorption of FR light [7].

In addition to photoconversion, the P_fr_ form can also fall back into the P_r_ form spontaneously, since the P_fr_ form has lower thermostability. This light-independent conformation change of the photoreceptor, also called dark reversion, was first suggested to function as an additional inactivation process by Eichenberg et al. [11]. It has been demonstrated by these authors that dark reversion beside being an intrinsic property of the molecule is also affected by cellular components and shows strong temperature dependence [12]. Whether the molecular dynamics of dark transition from P_fr_ to P_r_ are identical to and mediated by the same intermediate states as the light induced P_fr_ to P_r_ transition is not yet known.

Based on their mode of action and the light stability of the proteins, phytochromes can be divided into two classes. Type I phytochromes controlling the Very Low Fluence Responses (VLFR) and the far-red High Irradiance Responses (HIR) show rapid degradation of the P_fr_ form. Type II phytochromes are light stable and control Low Fluence Responses (LFR) and red light High Irradiance Responses. Using mutant analysis, it was shown that type I phytochromes are encoded by the *PHYA* gene and type II phytochromes by the *PHYB, PHYC, PHYD* and *PHYE* genes [6], [8], [13].

Physiological experiments showed that all these phytochromes sense not only the quality but also the quantity of light. Light quality sensing is explained by the formation of different amounts of the active P_fr_-form and for phyA, a light quantity sensing mechanism can also be envisaged. In this model the fast proteolytic degradation of phyA [14] and the EID1 dependent degradation of additional signal transduction components are likely to play major roles. EID1 is an F-box protein and mutations of the *EID1* gene lead to light hypersensitivity and a shift of the action spectrum from the far-red peak to a red peak [15], [16], [17].

In the case of phyB the capacity to monitor light quantity is less obvious [18], as phyB-mediated responses cannot be provoked by a single R light pulse that establishes saturating P_fr_ levels, whereas continuous red light elicits strong responses [19], [20]. Recent observations, however, suggest that dark-reversion [21], proteolytic degradation of the active phyB P_fr_ form [22] and formation of phyB-containing nuclear bodies [23] are important factors in mediating light quantity measurement by this photoreceptor.

Accordingly, expression of phytochrome cDNA in yeast cells and reconstitution of the holo-photoreceptor by addition of the chromophore have shown that all tested phytochromes exhibit partial dark reversion of P_fr_ to P_r_ after phototransformation of P_r_ to P_fr_ [24]. Recently, a slower but almost complete dark reversion of phyB has been measured *in vivo*, in phyB over-expressing lines in a phyA null background [21], whereas Oka et al. [25], [26] reported that the N-terminal PHY domain of phyB is involved in regulating dark reversion of the photoreceptor in transgenic plants. Interestingly, it has been demonstrated [21], [27] that dark reversion of phyB is at least partially regulated by its interaction with the response regulator ARR4. Thus it follows that regulation of dark reversion of phyB is likely to be a target for hormone-induced signalling pathways.

In etiolated seedlings, phyB is predominantly localized in the cytosol [28]. After transfer to light, nuclear import of phyB and formation of intra-nuclear speckles, also termed as nuclear bodies (NBs) have been described [28]. Surprisingly, these reactions show a strong light quality and quantity dependence, specific for the individual photoreceptors [20], [23], [28], [29,]. Although the precise biological function of the different types of phyB-associated NBs [29]–[32] is not fully understood, it is evident that the majority of phyB signalling mutants display aberrant NB formation [20], [29], [31]. Moreover, recent data described by Rausenberger et al. [23] suggest that, in contrast to phyB P_fr_ localized in the nucleoplasm, the NB-associated phyB P_fr_ conformers are prevented from fast dark-reversion.

The *phyB-401* mutant isolated by Kretsch et al. [33] shows extreme hypersensitivity to red light and displays strongly reduced reversibility by far-red light. The same authors reported that the phyB-401 photoreceptor exhibits a nearly complete lack of dark reversion but normal photoreversibility in yeast cells. The reduced dark reversion could, in principle, explain the extreme hypersensitivity to R light, but it is not sufficient to account for the poor far-red light reversibility of the inhibition of hypocotyl growth. To interpret the complex phenotype of the *phyB-401* mutant at molecular level we raised transgenic lines expressing the wild–type PHYB:GFP/YFP and mutant PHYB-401:GFP/YFP fusion proteins in *phyB-9* background. Next we measured (a) the rate of dark reversion and light induced photoconversion of the mutant phyB-401 and wild type phyB fusion proteins *in planta* and (b) analysed far-red reversibility of cellular and molecular events related to phyB action. Our data suggest that the complex phenotype of *phyB-401* can be best explained by assuming that the G to E amino acid change at position 564 inhibits dark reversion and at least partially uncouples photoconversion of the chromophore from the P_fr_-P_r_ conformational change of the holoprotein.

## Results

We have generated several independent transgenic lines expressing the 35S:PHYB-401:YFP and 35S:PHYB-401:GFP fusion proteins in *phyB-9* background. The primary transgenic plants were selfed, homozygous segregants were selected and lines displaying stable phenotype were multiplied for additional experiments. Next, the expression level of the PHYB-401:YFP or GFP fusion proteins was determined and compared to PHYB:GFP and PHYB:YFP in *phyB-9* and endogenous PHYB in WT backgrounds, respectively. The transgenic lines expressing the 35S:PHYB:GFP or YFP fusion protein used in this study had been isolated previously, they display stable phenotypes and fully complement the *phyB-9* mutant [20], [28]. We performed a series of western-blot hybridization experiments and selected one line for further experiments from each of the 35S:PHYB-401:YFP, 35S:PHYB-401:GFP, 35S:PHYB:GFP and 35S:PHYB:YFP lines. The selected transgenic lines over-express the fusion proteins 3-8 times as compared to endogenous phyB (for details see Table S1). Experiments described below were performed using these 4 selected lines. Data obtained using the YFP and GFP fusion proteins were comparable and did not differ significantly.

### Fluence rate response analysis of hypocotyl growth inhibition

**Figure 1:**
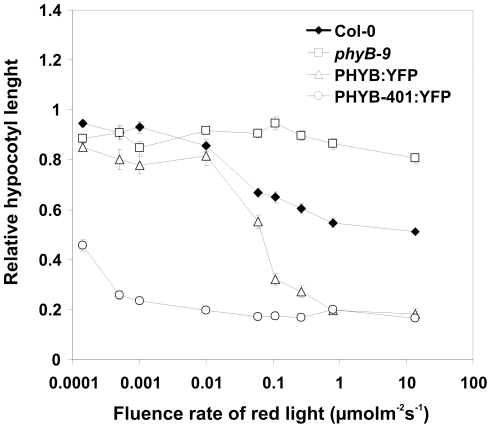
Fluence rate dependent hypocotyl growth inhibition of PHYB:YFP and PHYB-401:YFP seedlings. Wild-type Col-0 (filled diamonds), *phyB-9* mutant (empty squares) and *phyB-9* transgenic seedlings expressing the PHYB:YFP (empty triangles) and PHYB-401:YFP (empty circles) fusion proteins were grown for 4 days under different fluence rates of cR light. Hypocotyl lengths were measured and the relative hypocotyl lengths are shown. The experiments were repeated three times, error bars are also shown.

Figure 1 shows the fluence rate response curves obtained for WT (Col), *phyB-9* and transgenic PHYB-401:YFP and PHYB:YFP seedlings in cR. This figure illustrates that the transgenic line expressing the PHYB-401:YFP fusion protein displays extreme hypersensitivity to cR, since these seedlings reach saturation of the response already at approximately 0.001 µmol m^−2^ s^−1^. In contrast, the transgenic PHYB:YFP and the WT (Col) seedlings start responding at ∼0.01 µmol m^−2^ s^−1^ and show saturation around 1 and 10 µmol m^−2^ s^−1^, respectively. Transgenic lines expressing the PHYB-401:GFP fusion protein displayed similar hypersensitivity to R (Figure 1 and Figure S1A). We note that all seedlings expressing PHYB-401 and PHYB fusion proteins displayed normal etiolated phenotype as compared to WT seedlings (Figure S2). Taken together, these fluence rate measurements are in good agreement with the data reported by Kretsch et al. [33] and indicate that the PHYB-401:YFP and GFP fusion proteins affect R induced inhibition of hypocotyl elongation as expected.

### In vivo dark reversion kinetics of PHYB-401:YFP after saturating red light treatment

**Figure 2:**
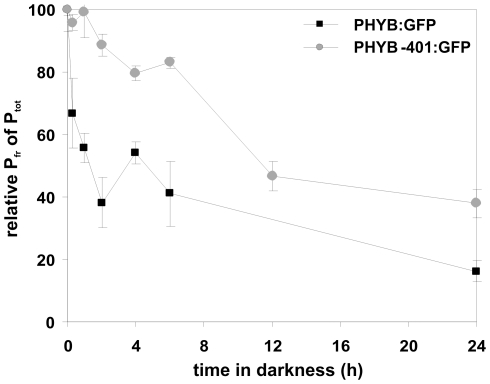
*In vivo* dark reversion rate of PHYB-401:GFP is slower compared to PHYB:GFP. Dark reversion kinetics of PHYB:GFP and PHYB-401:GFP fusion proteins, measured *in planta*, is shown. 4-day-old etiolated seedlings were irradiated 3 h with R (3 µmol m^−2^ s^−1^), returned to darkness and the amount of PHYB:GFP (black squares) and PHYB-401:GFP (grey circles) P_fr_ relative to P_tot_ was measured, as indicated, during a 24 h period.

Data reported by Kretsch et al. [33] demonstrated that the phyB-401 photoreceptor exhibits a reduced dark reversion *in vitro*. To confirm and extend this observation, we measured the dark reversion kinetics of the PHYB-401:GFP fusion protein *in planta*. To this end, 4-day-old seedlings were irradiated for 3 h with R (3 µmol m^−2^ s^−1^) and the amount of P_fr_ was measured directly with a dual-wavelength ratio spectrophotometer (RatioSpec) during the following dark period. For detailed information about the RatioSpec measurement see Materials and Methods. Figure 2 shows that *in vivo* dark reversion of PHYB:GFP has a half life time of around 1 h, which is in good agreement with previously published data [21], [23]. As expected, dark reversion of the PHYB-401:GFP expressing line displayed a different, strongly reduced reversion kinetics (Figure 2). This figure illustrates that after 12 h of darkness about 50%, whereas after 24 h darkness about 40% of the original amount of PHYB-401:GFP P_fr_ is still detectable. These data unambiguously demonstrate that the PHYB-401:GFP fusion protein, like the PHYB-401 protein *in vitro*, shows a strongly reduced dark reversion *in vivo*. This in turn results in an increased stability of the PHYB-401 P_fr_ form, which is especially important in continuous weak red light or after light pulses. Under these conditions, where only a low percentage of PHYB P_fr_/P_fr_ homodimers are generated [6], the enhanced stability of the P_fr_ form of the PHYB-401:YFP fusion protein can indeed result in an increased sensitivity, but it has little to do with the loss of reversibility response described by Kretsch et al. [33].

### In vivo photoconversion of PHYB and PHYB-401 P_fr_ to P_r_

**Figure 3:**
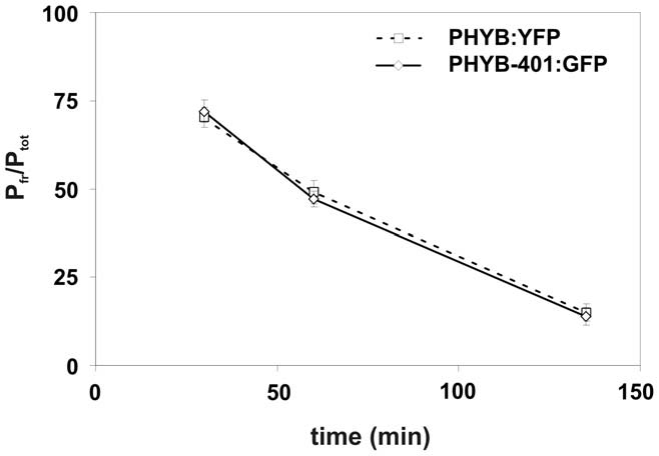
*In vivo* photoconversion. 4-day-old dark-grown PHYB:YFP and PHYB-401:GFP expressing seedlings were irradiated for 3 h with 40 µmol m^−2^ s^−1^ red light to convert PHYB and PHYB-401 to the P_fr_ form. Subsequently, seedlings were treated with 6 µmol m^−2^ s^−1^ far-red light (720 nm) on ice for 30 sec, 60 sec or 135 sec. P_fr_ and total phy content (P_tot_) was measured in dual-wavelength ratio spectrophotometer. The ratio of P_fr_/P_tot_ is plotted against the far red pulse duration. Error bars represent standard error.

Kretsch et al. [33] demonstrated that photoconversion of phyB-401 does not significantly differ from that of the wild type phyB *in vitro*. To evaluate whether this statement is valid *in vivo* we measured far-red light induced photoreversibility of PHYB:GFP and PHYB-401:GFP fusion proteins *in planta*. Photoconversion of PHYB-401:GFP and PHYB:GFP P_fr_ to P_r_ was determined as follows. 4-day-old etiolated seedlings were treated with red light for 3 h (40 µmol m^−2^ s^−1^) to convert all phyB P_r_ into P_fr_ form and simultaneously to degrade phyA. During the last 15 min of red light treatment seedlings were placed on ice and afterward all additional steps were carried out on ice to avoid dark reversion of the generated phyB P_fr_. After completing the red light treatment seedlings were irradiated with 6 µmol m^−2^ s^−1^ far-red light (720 nm) for 30 s, 60 s or 135 s and the P_fr_ and total phy levels were measured in dual-wavelength ratio spectrophotometer (Ratiospect). Figure 3 demonstrates that photoconversion of PHYB:GFP and PHYB-401:GFP P_fr_ to P_r_ is identical *in vivo*. These data suggest that aberrant/impaired photoconversion of the phyB-401 P_fr_ to P_r_ cannot be accounted for the loss of far-red reversibility of phyB-401 mutant regulated responses.

### Light quantity dependent nucleo-cytoplasmic partitioning of the photoreceptor:YFP fusion proteins in seedlings grown in cR

**Figure 4:**
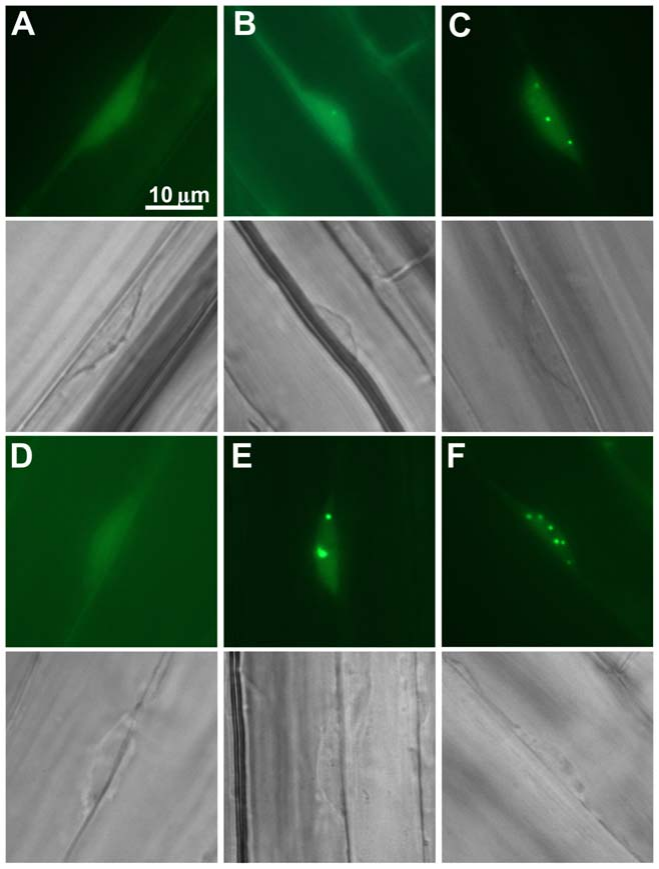
Subcellular distribution of PHYB:YFP and PHYB-401:YFP differs in seedlings irradiated with cR. Localization of PHYB:YFP (A, B, C) and PHYB-401:YFP (D, E, F) fusion proteins in 4-day-old seedlings grown in darkness (A, D), low-intensity (0.03 µmol m^−2^ s^−1^) (B,E) or high-intensity cR light (22 µmol m^−2^ s^−1^) (C, F) is shown. Below the fluorescence pictures of cells (A–F) the corresponding differential interference contrast microscopic images are also shown.

We examined the subcellular localization pattern of the PHYB-401:YFP and PHYB:YFP fusion proteins in transgenic seedlings grown for 4 days in darkness or at 0.03 µmol m^−2^ s^−1^ and 22 µmol m^−2^ s^−1^ intensities of cR. It is estimated that under these conditions the biologically active P_fr_ conformer represents ∼20 and >80% of the total phyB amount, respectively (for a detailed explanation for the calculation of the P_fr_/P_tot_ ratio see Material and Methods). PHYB:YFP displayed diffuse staining and was detectable in the nuclei and cytoplasm in dark-grown seedlings (Figure 4A). Figure 4B shows that illumination of PHYB:YFP seedlings with weak cR induces, in a low percentage of cells, the formation of a few small NBs. Irradiation with higher light intensity clearly promotes association of the PHYB:YFP fusion protein with NBs (Figure 4C). PHYB:YFP was still detectable in the cytoplasm after illumination with weak cR (Figure 4B), whereas the absence of cytoplasmic YFP signal in seedlings irradiated with high-intensity R light (Figure 4C) indicates an increased level of nuclear import of the PHYB:YFP. Intracellular distribution of PHYB-401:YFP was clearly different from that of PHYB:YFP. Figure 4E and F demonstrate that irradiations with low and/or high intensity cR uniformly induced formation of numerous large PHYB-401:YFP NBs, whereas, as shown in Figure 4D–F, cytoplasmic YFP fluorescence was detectable only in etiolated material. Taken together, these data indicate that nuclear import and formation of large PHYB-401:YFP associated NBs display increased sensitivity in cR as compared to PHYB:YFP.

### Kinetics of the formation, stability and photoreversibility of R pulse induced nuclear bodies containing PHYB-401:YFP and PHYB:YFP

**Figure 5:**
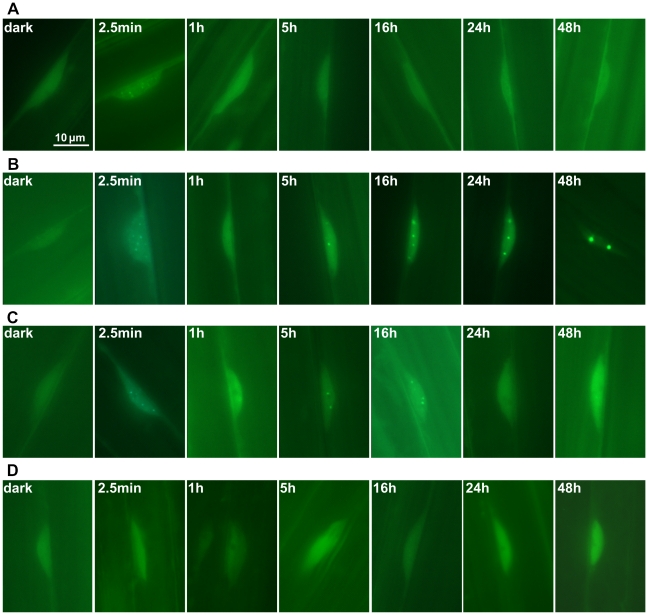
R pulse induced formation of PHYB-401:YFP nuclear bodies is not fully reversible by FR. (A) Localization of PHYB:YFP or (B) PHYB-401:YFP fusion proteins in 4-day-old seedlings grown in darkness, treated with a high intensity red light pulse (30 s, 70 µmol m^−2^ s^−1^) and returned to darkness. (C) Localization of PHYB-401:YFP or (D) PHYB:YFP fusion proteins in 4-day-old etiolated seedlings, irradiated with the same high-intensity red light pulse followed by a RG9 pulse (2 min, 40 µmol m^−2^ s^−1^) and then transferred to darkness. Localization of the fusion proteins was monitored at times after the light treatments indicated in the upper right corner of the pictures. Scale bar is shown.

First we tested the effectiveness of a single R pulse to induce the formation of phyB NBs. To this end we monitored cellular distribution of PHYB:YFP and PHYB-401:YFP in 4-days-old dark-grown seedlings that had been treated with a short R pulse (30 s, 70 µmol m^−2^ s^−1^) and then returned to darkness. In etiolated seedlings both PHYB:YFP (Figure 5A, D) and PHYB-401:YFP (Figure 5B, C) showed weak, diffuse nuclear staining. The applied high-intensity R pulse induced rapid formation of numerous, small NBs associated with PHYB:YFP or PHYB-401:YFP already within 2 minutes after the treatment. These NBs were short-lived and transient, as they were not detectable later than 1 hour after the R pulse (Figure 5A and B). As for PHYB:YFP, we could not detect NBs in samples monitored 5 h, 16 h, 24 h and 48 h after the treatment: the PHYB:YFP fusion protein remained uniformly distributed in the nucleoplasm (Figure 5A). In contrast, the PHYB-401:YFP-associated NBs reappeared in the nuclei within 5 h after the R pulse (Figure 5B). These newly formed, late NBs differed from the transient NBs detected shortly after the light treatment, i.e. they were less numerous but larger and, surprisingly, they were detectable and got even larger up to 48 h of darkness (Figure 5B). As only the P_fr_ conformer of phyB is known to be able to associate with NBs, these observations imply that the P_fr_ form of PHYB-401:YFP is more stable than that of PHYB:YFP. Furthermore the disappearance of early speckles and appearance of late NBs after 2–3 h in complete darkness, indicates that PHYB-401:YFP remains in the P_fr_ state in darkness for long time and that formation of PHYB containing late NBs takes several hours even in the continuous presence of P_fr_. This slow process can either be due to the slow modification of pre-existing NBs to allow interaction with PHYB P_fr_ or a slow modification of PHYB P_fr_ to facilitate interaction with preexisting NBs.

We also tested far-red light reversibility of this process. For these assays we employed a similar experimental setup, i.e. 4-day-old-etiolated seedlings were treated with a short R pulse (30 s, 70 µmol m^−2^ s^−1^) directly followed by a 2 min RG9 pulse (40 µmol m^−2^ s^−1^) and transferred to darkness. We found that the RG9 treatment prevented the appearance of R-induced early, transient PHYB:YFP NBs (Figure 5D **)**. In contrast, Figure 5C demonstrates that the formation of R-induced PHYB-401:YFP-associated NBs, early/transient as well as late/stable ones, was only partially affected by RG9. These nuclear complexes remained clearly detectable, although RG9 irradiation lowered the number of the early, transient and the newly appearing late PHYB-401:YFP NBs. As only the P_fr_ conformer of phyB is known to be able to associate with NBs, these experiments suggest that RG9-induced photoconversion of PHYB-401:YFP P_fr_ holoprotein to P_r_ is significantly impaired as compared to PHYB:YFP. We conclude this despite the fact that *in vivo* spectroscopic measurements of the P_fr_ to P_r_ photoconversion -monitoring the photoconversion of the chromophore - does not show any significant alteration (Figure 3).

### Characterization of the photoreversibility of cR-induced PHYB:YFP and PHYB-401:YFP nuclear bodies

**Figure 6:**
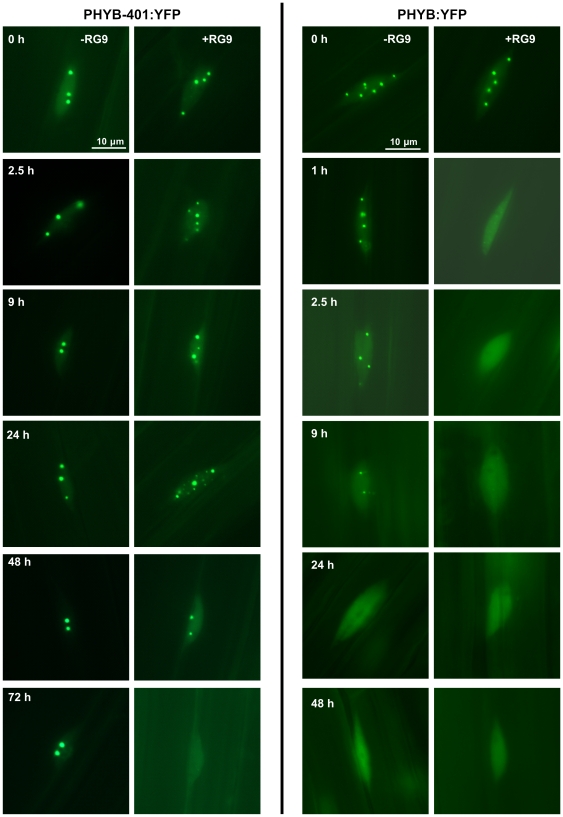
RG9 treatment is ineffective in depleting cR-induced PHYB-401:YFP NBs in darkness. Seedlings expressing PHYB-401:YFP or PHYB:YFP were grown for 3 days in darkness and on day 4 they were irradiated with cR (3 µmol m^−2^ s^−1^). After the 24 h R irradiation the seedlings were transferred to darkness (−RG9) or were treated with a RG9 pulse (40 µmol m^−2^ s^−1^) prior to transfer to darkness (+RG9). Subcellular localization of the PHYB-401:YFP and the PHYB:YFP fusion proteins were monitored after the light treatments. Sampling time (h) after the light treatments is indicated in the left panels. Scale bar is shown.

Rausenberger et al. [23] demonstrated that light treatments reverting P_fr_ back to the P_r_ form led to a fast depletion of stable phyB NBs. Thus we concluded that monitoring the depletion kinetics of cR-induced PHYB-401:YFP NBs after an RG9 pulse provides an alternative method for testing photoreversibility of these nuclear complexes. To perform these studies, 3-day-old etiolated seedlings were irradiated with cR light (3 µmol m^−2^ s^−1^) for 24 h and then either transferred to darkness, or irradiated with an additional RG9-pulse (2 min, 40 µmol m^−2^ s^−1^) prior to transfer to darkness. Localization of the PHYB-401:YFP fusion protein was analyzed immediately after the light treatments and during the following dark period, up to 6 days. Figure 6 (left panel) shows that without the RG9 pulse the PHYB-401:YFP NBs are stable in darkness, since their number and size do not change significantly up to 72 h. This figure also shows that treatment with RG9 pulse did not deplete the PHYB-401:YFP NBs up to 48 h after the pulse but led to the appearance of smaller, more numerous NBs. These observations are in sharp contrast to data reported by Rausenberger et al. [23], who showed that cR-induced PHYB:YFP NBs were no longer detectable after 9 h in darkness and RG9 treatment significantly accelerated depletion of these nuclear structures that became undetectable within 1 h after the RG9 treatment. Finally, we note that the RG9-pulse applied during these assays did not induce NB formation or any physiological response in seedlings expressing the PHYB-401:YFP fusion protein. Taken together, these and the data shown in Figure 5C indicate that the RG9-generated P_r_ form of PHYB-401:YFP is still capable of associating with NBs, this P_r_ form is relatively stable and its properties resemble that of the biologically active P_fr_ form of PHYB:YFP.

### Comparison of the signalling efficiency of PHYB:GFP and PHYB-401:GFP

**Figure 7:**
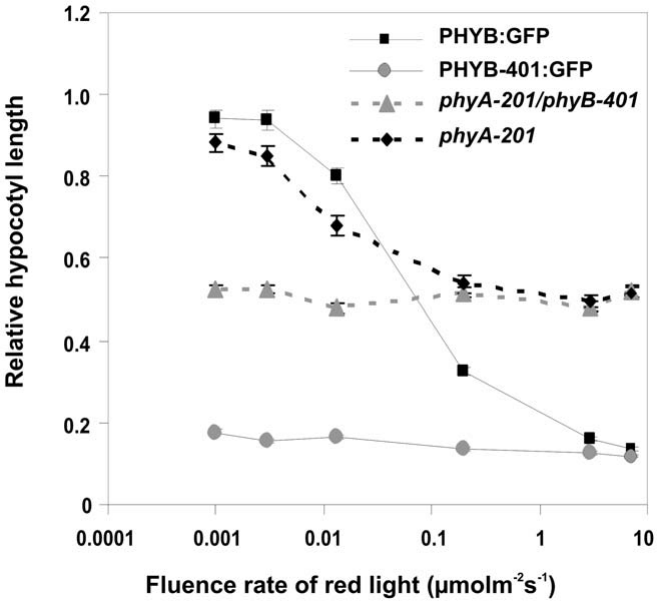
Signalling efficiency of phyB-401 does not differ from phyB in saturating cR. Mutant *phyA-201* (black diamonds, dashed line), *phyA-201/phyB-401* (grey triangles, dashed line) as well as transgenic PHYB:GFP (black squares) and PHYB-401:GFP (grey circles) seedlings in *phyB-9* background were grown for 4 days under different intensities of cR. Hypocotyl lengths were measured and the relative hypocotyl lengths are shown. Experiments were repeated three times, error bars are shown.

Beside the decreased rate of dark reversion, the G to E change at position 564 may influence interaction of the mutant photoreceptor with components of the signal transduction cascade and thereby contribute to the dramatic hypersensitivity of the *phyB-401* mutant and the PHYB-401:GFP seedlings as compared to WT (Figure 7). Since it has been shown that the total amount of phyB is the limiting factor for maximal signal strength [23], comparison of fluence rate responses of lines containing different levels of total phyB can provide additional information concerning the mechanism underlying the hypersensitive phenotype of the *phyB-401* mutant. To achieve this, we measured fluence rate dependent inhibition of hypocotyl growth in lines containing different amounts of phyB (in *phyA-201* and 35S:PHYB:GFP in *phyB-9*, respectively) and mutant phyB (in *phyB-401/phyA-201* and 35S:PHYB-401:GFP in *phyB-9* backgrounds, respectively). Over-expression levels are shown in Table S1. Figure 7 shows the relation between the amount of the photoreceptor and the maximal response/signal for the mutant phyB (in *phyB-401/phyA-201* and PHYB-401:GFP in *phyB-9)* and wild-type phyB (in *phyA-201* and PHYB:GFP in *phyB-9*). This figure demonstrates that under saturating light conditions (10 µmol m^−2^ s^−1^ red light) the amount of phyB or phyB-401 P_fr_ neither in *phyA-201* nor *in phyA-201/phyB-401* background is sufficient to induce maximal physiological response in contrast to the transgenic lines expressing the PHYB:GFP and PHYB-401:GFP in *phyB-9* background. Because at 10 µmol m^−2^ s^−1^ fluence rate the amounts of phyB and phyB-401 and PHYB:GFP and PHYB-401:GFP P_fr_ are pairwise identical we conclude that the phyB-401 P_fr_ molecule is not more efficient than the wild-type photoreceptor.

However, the extreme hypersensitivity for red light induced physiological responses displayed by the *phyA-201/phyB-401* and PHYB-401:GFP seedlings makes it difficult to asses the signalling efficiency under non-saturating conditions. To determine this by using the hypocotyl growth inhibition as a read-out we have to know the exact amount of phyB and phyB-401 P_fr_ present. This parameter we can estimate only with large uncertainty because of the altered dark reversion and finding shown in Figure 6 which indicates that the cycled P_r_ form of phyB-401 may display biological activity. To overcome this difficulty we compared signalling efficiency of the wild type and the mutant phyB-401 photoreceptors by other experimental approaches described below.

### Kinetics of signalling initiated by PHYB:YFP and PHYB-401:YFP

**Figure 8:**
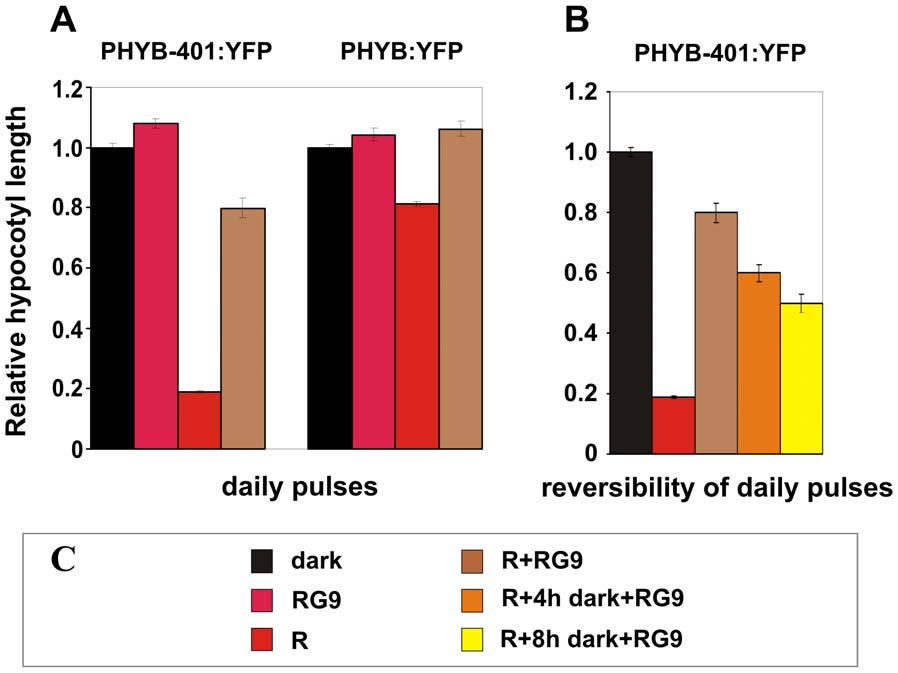
Hypocotyl growth inhibition of PHYB-401:YFP seedlings induced by R pulses is conditionally photoreversible. (A) PHYB-401:YFP and PHYB:YFP transgenic seedlings were grown for 4 days in darkness (black bars) or treated daily with a RG9 pulse (2 min, 40 µmol m^−2^ s^−1^, dark-red bars), a R light pulse (30 s, 70 µmol m^−2^ s^−1^, red bars), or immediately after the R pulse with a RG9 pulse (2 min, 40 µmol m^−2^ s^−1^, brown bars) and returned to darkness. (B) PHYB-401:YFP seedlings were grown for 4 days in darkness (black bar) or treated daily with a R light pulse (30 s, 70 µmol m^−2^ s^−1^, red bar) or after the R pulse were treated immediately with a RG9 pulse (2 min, 40 µmol m^−2^ s^−1^, brown bar) and returned to darkness. Alternatively, after the R pulse treatment seedlings were returned to darkness for 4 h (orange bar) or 8 h (yellow bar), then irradiated with a RG9 pulse, and again returned to darkness until the next pulse treatment. (C) Symbols illustrate the various light pulse treatments applied. Hypocotyl lengths of seedlings were measured and relative hypocotyl lengths are shown. Error bars illustrate the standard error of the mean.

As RG9 pulses were shown to revert hourly R pulses in WT but not in the *phyB-401* mutant [33] we tested the reversibility of daily applied R pulses. Figure 8A shows that under these conditions seedlings expressing the wild-type PHYB:YFP display a weak hypocotyl growth inhibition response to daily applied R pulses, whereas the PHYB-401:YFP seedlings show an almost saturated growth inhibition. We note that the observed strong effect of daily red light pulses is in good agreement with the reduced dark reversion kinetics of PHYB-401 and clearly demonstrates the physiological consequence of the increased stability of the P_fr_ form. The same figure, however, also shows that (i) the effect of daily R pulses on hypocotyl growth of the PHYB-401:YFP seedlings can be reverted (to about 80%) by an additional RG9 pulse (ii) which alone was not inductive. We also determined the kinetics of the reversibility of PHYB-401:YFP-controlled inhibition of hypocotyl growth. To this end we grew PHYB-401:YFP seedlings as described above but included 4 and 8 h dark intervals between the inductive R and the reverting RG9 pulses. Figure 8B illustrates that the PHYB-401:YFP-induced response can be partially reversed by far-red pulses applied leading to 60% and 50% reduction of hypocotyl length, respectively. These data fit well the kinetics of the loss of reversibility of wild-type phyB published recently by Hennig et al. [34]. Taken together, we conclude that (i) the kinetics of signal transduction initiated by the mutant phyB-401 photoreceptor and wild-type phyB do not differ significantly, and that (ii) the loss of photoreversibility of the R pulse induced hypocotyl growth described by Kretsch et al. [33] is not caused by an accelerated signal transduction operating in the *phyB-401* seedlings.

### Fluence rate dependence of light pulse induced accumulation of CAB2 mRNA

**Figure 9:**
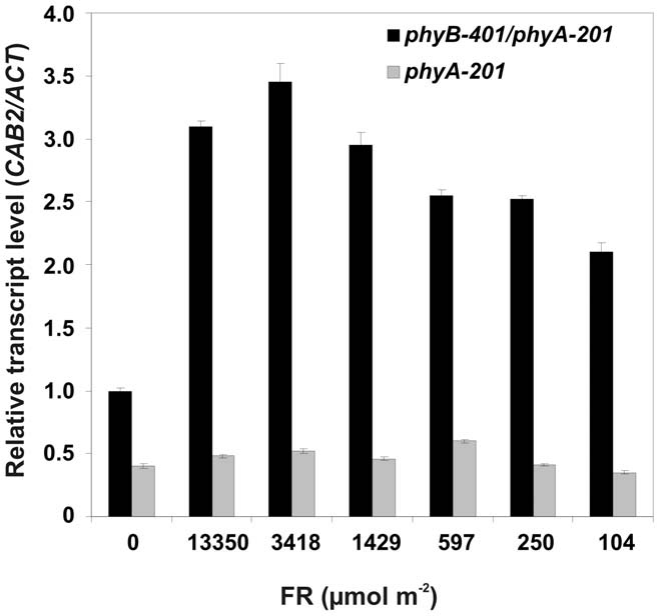
FR induces *CAB2* mRNA accumulation in *phyB-401/phyA-201*seedlings. *phyB-401/phyA-201* (black bars) and *phyA-201* (grey bars) seedlings were grown for 4 days in darkness before onset of light treatments and were analysed by real time PCR for *CAB2* and *ACTIN* mRNA accumulation after additional dark incubation of 6 h. 5 min FR light pulses of the indicated total fluences were applied before dark transfer. Relative transcript levels of *CAB2/ACTIN* are shown, the value of the *phyB-401/phyA-201* dark sample was set to one. Error bars illustrate the standard error of the mean.

To compare the sensitivity of PHYB and PHYB-401 photoreceptors at the molecular level we tested the *CAB2* mRNA accumulation after an inductive far red light pulse. 4-day-old etiolated seedlings were irradiated for 5 min with far red light of variable fluence rates to establish fluences between 104 and 13350 µmol m^−2^. Figure 9 shows that these fluences were not inductive in *phyA-201* but they caused significant elevation of *CAB2* mRNA levels in *phyA-201/phyB-401*. This figure also shows that maximal induction of *CAB2* mRNA was detected at 3418 µmol m^−2^ fluence. These data demonstrate the hypersensitivity of the PHYB-401 because at least at the higher fluences the same P_fr_ levels should be established for PHYB and PHYB-401. The unexpected fluence dependence of the *CAB2* mRNA induction by a far-red light pulse indicates that the photochemical cycling of the PHYB-401 photoreceptor produces an active signalling component.

## Discussion

The intragenic *phyB-401* point mutant reported by Kretsch et al. [33] displays extremely enhanced light sensitivity and loss of photoreversibility of hypocotyl growth inhibition to hourly R light pulses. This phenotype is unique among the numerous phyB intragenic mutants isolated to date yet it is still unclear how the G to E amino acid substitution at position 564 modifies phyB signaling at the molecular level. Here we report about a broad array of experiments performed with PHYB-401:YFP and GFP expressing transgenics that allowed us to confirm and extend results reported by Kretsch et al. [33]. We show that the 35S: PHYB-401:YFP and GFP transgenic lines mimic the phenotype of the original *phyB-401* mutant, display extreme hypersensitivity to cR (Figure 1 and Figure S1A) and to cFR (Figure S1B). We also demonstrate that the PHYB-401:YFP fusion protein has a reduced dark reversion rate *in vivo* (Figure 2), whereas its *in vivo* photoconversion rates (Figure 3), its degradation in cR (Figure S3) and signalling efficiency under saturating light conditions (Figure 7) are identical to PHYB:YFP. Importantly, we found that hypocotyl growth inhibition induced by daily applied R pulses (in contrast to hourly R pulses) is photoreversible in PHYB-401:YFP seedlings (Figure 8A), which in turn allowed us to document, by increasing the interval between the inductive R and the inhibitory RG9 pulses, that signaling launched by the mutant photoreceptor is not faster than that initiated by wild-type phyB (Figure 8B).

Monitoring light-induced changes of the nucleo/cytoplasmic distribution of PHYB and PHYB-401:YFP and/or GFP provided essential information for identifying a novel feature of the mutant photoreceptor and for suggesting a plausible explanation for the appearance of the unique phenotype. Our data clearly demonstrate that PHYB-401:YFP NBs are more stable in darkness (Figure 5B and Figure 6), RG9 light is ineffective to prevent appearance of R-induced PHYB-401:YFP NBs (Figure 5C) and/or to induce rapid depletion of PHYB-401:YFP NBs formed in cR (Figure 6, left panel) as compared to PHYB:YFP (Figure 5A, Figure 6, right panel). As PHYB NBs were shown to contain phyB P_fr_ [22], and phyB-401 shows normal spectral photoconversion (Figure 3], the data described above indicate that FR treatment produces a PHYB-401:YFP P form, designated P_r_*, that resembles P_fr_. In contrast to this the *in vivo* spectroscopic measurements show that photoconversion of the chromophore - as measured by *in vivo* spectroscopy - is normal. This P_r_* form is biologically active (Figure 9 and Figure S1B), it persists for some hours (Figure 5B and Figure 6) and it slowly falls back to P_r_ by a mechanism not yet elucidated. The kinetics of this process cannot be precisely estimated, however, our data show that the life time of P_r_* must be longer than one hour as hourly red light pulses are not reversible by far-red light. The reversibility of daily pulses and the persistence of the nuclear bodies suggest that the life time of P_r_* should be less than 8 hours. The precise calculation of the life time is not possible as the signaling efficiency of P_r_* compared to P_fr_ is unknown.

Our data demonstrate that such a P_r_* signaling competent can also be produced by far-red light pulses. The level of P_r_* will be elevated with increasing fluence, whereas the P_fr_ level will remain constant after the photoequilibrium is reached. Data obtained by the *in vivo* photoconversion measurements (Figure 3) allowed us to estimate that 90% saturation of photoequilibrium will be reached at 500 µmol m^−2^ s^−1^. Thus the fluence rate dependence of *CAB2* mRNA induction by far-red light pulses supports the hypothesis of the formation of a signaling P_r_*. The hypersensitivity of inhibition of hypocotyl growth of PHYB-401:YFP under cFR also cannot be explained by simply altered dark reversion of the mutant photoreceptor, because even in the absence of any dark reversion far-red light can only produce P_fr_ level up to 2–3%. (Figure S1B). The strong fluence rate dependency of this response suggests that this unexpected sensitivity of *phyB-401* mutant to continuous far-red light is mediated by photochemical cycling of the PHYB-401 photoreceptor. Under such conditions the P_r_* will be accumulated in a fluence rate dependent manner.

This hypothesis and the reduced dark reversion rate of the phyB-401 protein satisfactorily explain the extreme light sensitivity in weak cR and the loss of reversibility of hypocotyl growth inhibition to hourly R/FR pulses [33]. In weak cR, dark reversion of P_fr_ is the major regulator of P_fr_ levels. Under this condition only a low percentage of P_fr_/P_fr_ PHYB homodimers are generated [6], thus the low dark reversion rate leads to an enhanced stability of the PHYB-401:YFP P_fr_, which then results in an increased sensitivity. We note that dark reversion of PHYB-401:YFP could also generate a P_fr_-like P_r_* conformer, which then would further enhance the probability of the formation of the presumably active P_fr_/P_r_* dimers. This will also contribute strongly to the extreme enhanced responsiveness of PHYB-401:YFP or GFP to very low red light fluence rates under continuous irradiation (Figure 1 and 7). As far as the loss of photoreversibility of hypocotyl growth inhibition of the phyB-401 mutant is concerned [33], formation of the relatively long lived, biologically active phyB-401 P_r_* (Figure 5C, Figure 6) readily explains why hourly given RG9 pulses are ineffective in blocking hourly R-induced hypocotyl growth inhibition and why the phyB-401 does respond strongly to far-red light (Figure 9 and Figure S1B). However, we note that the proposed P_r_* form is likely to be less stable than the P_fr_ form. Data shown in Figure 8B support this conclusion by showing that hypocotyl growth inhibition of PHYB-401:YFP seedlings displays a significant, about 70% reversibility when R/RG9 pulses are applied at 24 h intervals.

The phenotype displayed by *phyB-401* mutant is sharply different from that of the constitutively active PHYB^Y276H^ mutant described by Su and Lagarias [35] and Hu et al. [36]. These authors showed that the universally conserved GAF domain Tyr residue, with which the chromophore is intimately associated, performs a critical role in coupling light perception to signal transduction. In other words, this particular amino acid substitution confers a conformation that mimics the photoactivated P_fr_ form of phyB in the absence of light in a chromophore-dependent fashion. Consequently, the PHYB^Y276H^ mutant displays de-etiolated phenotype in dark and fluence rate independent hypocotyl growth inhibition in light, whereas the PHYB-401:YFP seedlings display normal etiolated phenotype (Figure S2).

It is of interest to understand at the structural level why the G to E amino acid change at position 564 in the PHY domain affects photoconversion of the P_fr_ form to P_r_ so drastically. Crystal structure analysis of the Synechocystis phytochrome (Cph1) [37] showed that the PHY domain contains an additional unique feature, a tongue-like protrusion that is present in all phytochrome classes. According to Essen and colleagues [37] the tongue lies on the protein surface, it interacts with the GAF domain, seals the chromophore pocket and thereby ensures close packing of the chromophore and stabilization of the photoactivated P_fr_ state of phytochromes. The authors postulate that the very close packing of the chromophore may play a role in passing the chromophore's conformational change onto the protein, leading to the light-driven conformational change of phytochrome proteins and the transduction of the light signal. A sequence comparison of Arabidopsis phytochrome B and Cph1 shows that the G to E mutation of phyB-401 lies exactly in the conserved WGG motif of the tongue. Thus one can speculate that replacing a small G residue with a bulky polar E residue could disturb exactly that part of the phytochrome structure that couples the photoconversion of chromophore and protein. Thus it is possible that the mutation changes the conformation of the tongue-like protrusion in such a way that the first light switch from the inactive P_r_ to the active P_fr_ leads to a change in the holoprotein structure that reduces the dark reversion as well as the light-dependent conformational change back to the inactive state. If the chromophore's conformational change is uncoupled from the protein after the first switch, then the *phyB-401* mutant can hardly be switched off again by light. After reverting light treatments the mutant photoreceptor stays in an active form with the chromophore in its P_r_ state, while the protein possesses a P_fr_-like structure, thus it is still capable of light signalling. Only a slow reversion back to the real inactive P_r_ state (Figure 8B) can switch off the signalling again.

We have attempted to obtain direct biochemical evidence for demonstrating existence of the hypothesized phyB-401 P_r_* conformer. As our data indicated above the P_r_* conformer is clearly capable of signalling. Accordingly, we argued that this putative conformer should also be capable of binding to PIF3. To this end we have determined and compared binding of PIF3 to WT phyB and mutant phyB-401 P_fr_ and P_r_ proteins by yeast two-hybrid assays after different light treatments as shown in Figure S4. The ultimate goal of these studies was to demonstrate *in vitro* that FR treatment leads to the production of phyB-401 P_r_* thus reversion of PIF3 binding to the P_fr_ conformer of phyB-401, in contrast to that of phyB, shall not be complete. However, Figure S4 shows that capacity of P_fr_ forms of phyB and phyB-401 to bind PIF3 is approximately identical and that FR irradiation applied after the inductive R pulse completely abolishes binding of PIF3. Judicious interpretation of the data however suggests that these results should not be considered as a decisive proof for the non-existence of the putative phyB-401 P_r_* conformer. There are two major caveats of this experimental approach. First we perform these assays under saturating conditions and the sensitivity of such an assay is obviously limited. Second, we do not have reliable information about the stability of the hypothetical P_r_* *in vitro* as compared to *in vivo*. This latter one is especially a critical factor as the kinetics of accumulation of the hypothetical P_r_* conformer is likely to be different in the cellular environment *in planta* and yeast two-hybrid assays.

Taken together, data presented in this paper and elsewhere [24], [25] adequately support the importance of the PHY domain in regulating stability, i.e. dark reversion of the P_fr_ form. The ultimate proof for the mechanism by which it mediates passing the conformational change of the chromophore onto the protein could, however, only be obtained after the resolution of the 3D structure of the full phytochrome-B.

## Materials and Methods

### Plant material and growth conditions

Seeds were surface sterilized and sown on Petri-dishes (9 cm diameter) containing 4 layers of filter paper (Macherey-Nagel, Germany) and 4.7 ml distilled water. After stratification (three days at 4°C in darkness) uniform germination was induced by a 4 h white light treatment at 22°C. Afterwards seedlings were grown at 22°C in darkness for four days or subjected to various light treatments as described in the text. Transgenic plants expressing the 35S: PHYB:YFP and 35S:PHYB-401:YFP and GFP transgenes were generated in *phyB-9* [38] backgrounds, as described [19], [30]. The *phyB-401* mutant was isolated in Landsberg erecta background [33].

### Light treatments and hypocotyl length measurement

Dark-grown seedlings were handled under dim-green safelight [19]. For light pulse treatments and fluence rate response curves modified Leitz Prado 500-W universal projectors (Leitz, Wetzlar, Germany) were used. Red light was obtained by using KG65 filters (λmax = 650 nm; Blazers, Liechtenstein) and far-red light was generated by filtering with an RG9 filter (λmax = 775 nm; Schott, Germany) which adjusts 99.9% P_r_ [33]. Unless otherwise noted, all other red light treatments were done in a red light field (3 or 22 µmol m^−2^ s^−1^) of fluorescent tube lamps (Philips TL 40 W/15) filtered with Plexiglas 501/3 (Röhm und Haas, Darmstadt, Germany). Hypocotyl length of 4-day-old seedlings treated with various light programs was measured by using ImageJ software (http://rsb.info.nih.gov/ij).

### Epifluorescent and light microscopy

For epifluorescent and light microscopy, seedlings were transferred to glass slides under dim-green safelight and analyzed with an Axioskop microscope (Zeiss, Oberkochem, Germany). Excitation and detection of YFP were performed with specific YFP filter (AHF Analysentechnik, Tubingen, Germany). Each experiment was repeated at least three times using at least five seedlings in which we monitored minimum 200 nuclei in each single experiment.**** Representative cells were documented by photography with a digital Axiocam camera system (Zeiss). Photographs were processed for optimal presentation using the Photoshop 7.0 (Adobe Systems Europe, Edinburgh, UK) and MS Office 97 (Microsoft, Redmond, WA) software packages.

### In vivo spectroscopy

Dark reversion and photoconversion of transgenic PHYB-GFP and PHYB-401:GFP was measured in intact seedlings in a dual-wavelength ratio spectrophotometer (Ratiospect) [23], [39]. Seedlings were irradiated with saturating 3 h R to create maximal P_fr_ levels and to degrade light-labile phyA. The complete loss of phyA signal after this treatment was confirmed in *phyB-9* mutant seedlings (data not shown). For measurements of dark reversion, seedlings were either directly analyzed in the Ratiospect or transferred to darkness for measurement of P_fr_ levels in prolonged dark incubation. For photoconversion analysis, seedlings were placed on ice during the last 15 min of the R treatment and all later steps to avoid dark reversion. After R irradiations and before measurements seedlings were subjected to 30 s, 60 s or 135 s far-red light pulses (720 nm; 6 µmol m^−2^ s^−1^).

The total amount of phytochrome (P_tot_) was measured by six times alternate irradiation with far-red and red light followed by absorption measurements that gave the relative amount of photo-convertible phytochrome Δ (ΔA) of the sample. The absorption difference in the sample between the baseline at the beginning of spectroscopy and the first far-red light treatment 25 in the Ratiospect gave the P_fr_ level of the sample relative to P_tot_. For each measurement around 300 seedlings were used and fresh weight was determined directly before the measurement to normalize Δ (ΔA) between the samples. Each time point was measured at least three times, error bars indicate standard error.

### Calculation of P_fr_/P_tot_ ratio under continuous irradiation

Jabben *et al* [40] reported that the P_fr_/P_tot_ ratio measured in etiolated mustard seedlings is fluence rate dependent. To account for this phenomenon they hypothesized that an unknown reaction inducing conversion of P_fr_ to P_r_ in dark must exist. This process called dark-reversion was shown to enhance conversion of P_fr_ to P_r_, especially at low light. The ratio P_fr_/P_tot_ is k_1_/(k_1_+k_2_+k_r_) whereby k_1_ and k_2_ are the rate constants for P_r_ to P_fr_ and P_fr_ to P_r_ photoconversion, respectively. k_r_ is the rate constant for dark reversion. From Figure 3 k_1_ and k_2_ is estimated to be 0.008 s^−1^ and 0.002 s^−1^ at 6 µmol m^−2^ s^−1^. k_r_ is calculated from Figure 2 (k_r_ = 0.00013 s^−1^) if a first order dark reversion reaction is assumed.

### Protein extraction, protein gel blotting and immunodetection

To analyze cR-induced degradation of phyB 3-day-old dark-grown seedlings were irradiated up to 24 h in 3 µmol m^−2^ s^−1^red light and harvested together with a non-irradiated control at the end of irradiation period. Protein extraction and protein gel blotting was performed as described [30]. Immunodetection of phyB was performed using the monoclonal phyB antibody B6-B3 [41]. Horseradish-peroxidase-coupled anti mouse antiserum (Vector Laboratories) was used as secondary antibody. Development of the blot was done with the Phototrope Star Detection Kit (New England BioLabs). Quantification of phyB signals was carried out using the ImageJ software (http://rsbweb.nih.gov/ij/) and the gel analysis tools (described in the user manual: http.//rsbweb.nih.gov/ij/docs/user-guide.pdf). Linearity of the signal was ensured by the use of internal standards.

### Analysis of RNA accumulation by real-time RT-PCR

Far-red light treatments were carried out in a far-red light field for 5 min with varying intensities to obtain the aimed total fluences. Total RNA from Arabidopsis seedlings was isolated with Plant RNA reagent (Invitrogen) according to the manufacturer's protocol. Purification and on-column DNaseI digestion was performed by using the RNeasy mini Kit from Qiagen (Hilden, Germany). After first-strand cDNA synthesis, the RT-PCR was accomplished by using an ABI Prism7300 (Applied Biosystems). FAM- and JOE-reporter dyes (5′end) were used for the TaqMan probes (see below). A standard curve method was used for the calculation of relative transcript quantities [42]. Data were normalized to the corresponding *ACTIN1* mRNA levels; *phyB-401/phyA-201* relative dark levels were set to one. Each time point was measured at least in two biological replicas, error bars indicate standard error of technical replicas.

Sequences of primers and probes used for Real-Time RT-PCR were as follows: *ACTIN1* (forward: 5′- GGCTCCAAGCAGCATGAAG-3′; reverse: 5′- ACCCTCCAATCCAGACAGAGTATT-3′; probe: 5′JOE- CAAAGTCGTTGCCCCTCCAGAGAGG -3′BHQ1) and *CAB*2 (forward: 5′- GAGAGGCCGAGGACTTGCTT-3′; reverse: 5′- CTCTGGGTCGGTAGCCAAAC-3′; probe: 5′FAM- ACCCCGGTGGCAGTTTCGACC -3′TAMRA).

### Yeast two-hybrid assay

Quantitative yeast two-hybrid assay was performed as previously described [43].

## Supporting Information

Figure S1 **Fluence rate dependent hypocotyl growth inhibition of seedlings expressing PHYB and PHYB-401 fusion proteins.** Wild-type Col-0 (filled diamonds), *phyB-9* mutant (empty squares) and transgenic seedlings expressing PHYB:GFP or PHYB:YFP (empty triangle) and PHYB-401:GFP or PHYB-401:YFP (empty circle) fusion proteins in *phyB-9* (empty circle) were grown for 4 days under different fluence rates of cR (A) or cFR (B) light. Hypocotyl lengths were measured and relative hypocotyl lengths are shown. Each time point was measured at least three times, error bars indicate standard error.
(TIF)

Figure S2 **Overexpression of the PHYB-401:YFP fusion protein does not affect skotomorphogenesis of the transgenic seedlings.** Arabidopsis seedlings were germinated according to the standard protocol (after 3 days stratification at 4°C seeds were imbibed and treated with 4 h white light) and then grown for 4 days in darkness. Phenotypes of seedlings expressing the PHYB:YFP (A) and PHYB-401:YFP (B) fusion proteins in *phyB-9* background and those of the wild-type Col-0 (C) and *phyB-401/phyA-201* (D) double mutant are shown.
(TIF)

Figure S3 **Degradation kinetics of the mutant and wild-type phyB do not differ significantly in cR.** Accumulation levels of phyB and phyB-401 in *phyA-201* background were analyzed by western blot hybridization using the monoclonal antibody B3B6 [41]. Degradation of the native phyB (A) and mutant phyB-401 protein (B) is shown in *phyA-201* seedlings that were grown 4 days in darkness (d) and then irradiated with R light for 0, 3 and 24 h. The actin signals illustrate loading, the diagram (C) shows quantification of several western blots, error bars indicate standard error.
(TIF)

Figure S4 **Interaction of PIF3 with phyB and with phyB-401 is identical.** Liquid overnight cultures of yeast cells co-expressing the indicated proteins fused to the GAL4 activation (AD) or DNA-binding (BD) domain were treated with 5 min red light pulse (R) which was followed by a 5-min-long far-red light pulse (FR) in the indicated cases. After the given light pulses the cultures were incubated in the dark for the indicated time (between 2-6 h). Subsequently the ß-Galactosidase activity was determined. Error bars represent standard error of the mean.
(TIF)

Table S1 **Comparison of the expression levels of PHYB and PHYB-401 fusion proteins to the endogenous phyB and phyB-401 photoreceptors.** Accumulation levels of phyB, phyB-401 and those of the PHYB and PHYB-401 fusion proteins were analyzed by western blot hybridization using the monoclonal antibody B3-B6 [41]. Western blot hybridization assays were quantified (as described in Materials and Methods section) and expression levels of the PHYB and PHYB-401 fusion proteins in *phyB-9* background, the native phyB in Col-0 and *phyA-201* (*L. erecta*) backgrounds as well as the phyB-401 protein in *phyA-201* (*L. erecta*) background were compared. Fold expression levels normalized to endogenous phyB in Col-0 are shown.
(TIF)
