# Supplementary figures and images for: Altered Dark- and Photoconversion of Phytochrome B Mediate Extreme Light Sensitivity and Loss of Photoreversibility of the phyB-401 Mutant
Source: PLoS One. 2011 Nov 3;6(11):e27250. doi: 10.1371/journal.pone.0027250 (PMC3207837; doi:10.1371/journal.pone.0027250)

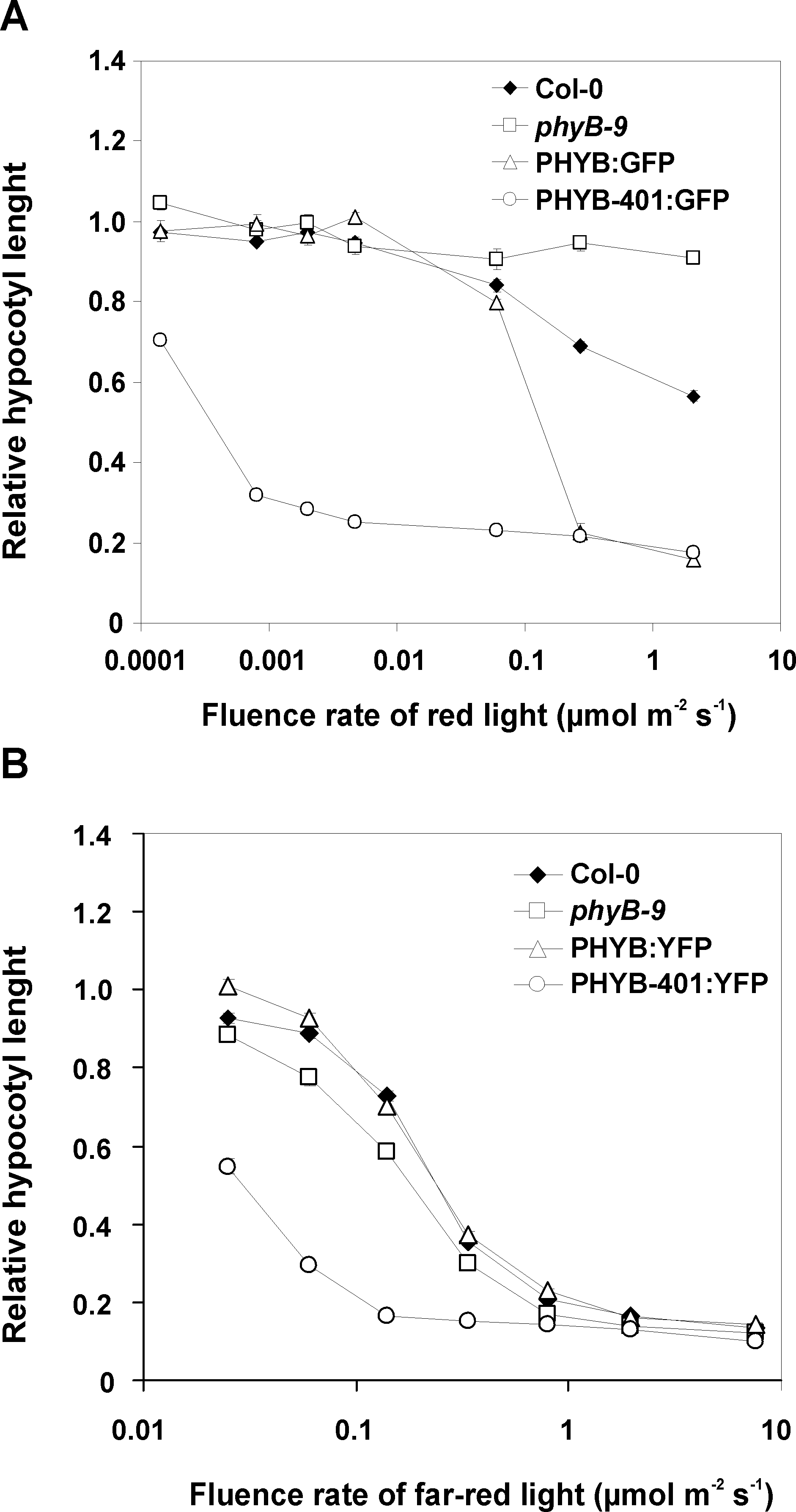

Supplement: Figure S1 — Fluence rate dependent hypocotyl growth inhibition of seedlings expressing PHYB and PHYB-401 fusion proteins. Wild-type Col-0 (filled diamonds), phyB-9 mutant (empty squares) and transgenic seedlings expressing PHYB:GFP or PHYB:YFP (empty triangle) and PHYB-401:GFP or PHYB-401:YFP (empty circle) fusion proteins in phyB-9 (empty circle) were grown for 4 days under different fluence rates of cR (A) or cFR (B) light. Hypocotyl lengths were measured and relative hypocotyl lengths are shown. Each time point was measured at least three times, error bars indicate standard error. (TIF) [file pone.0027250.s001.tif]

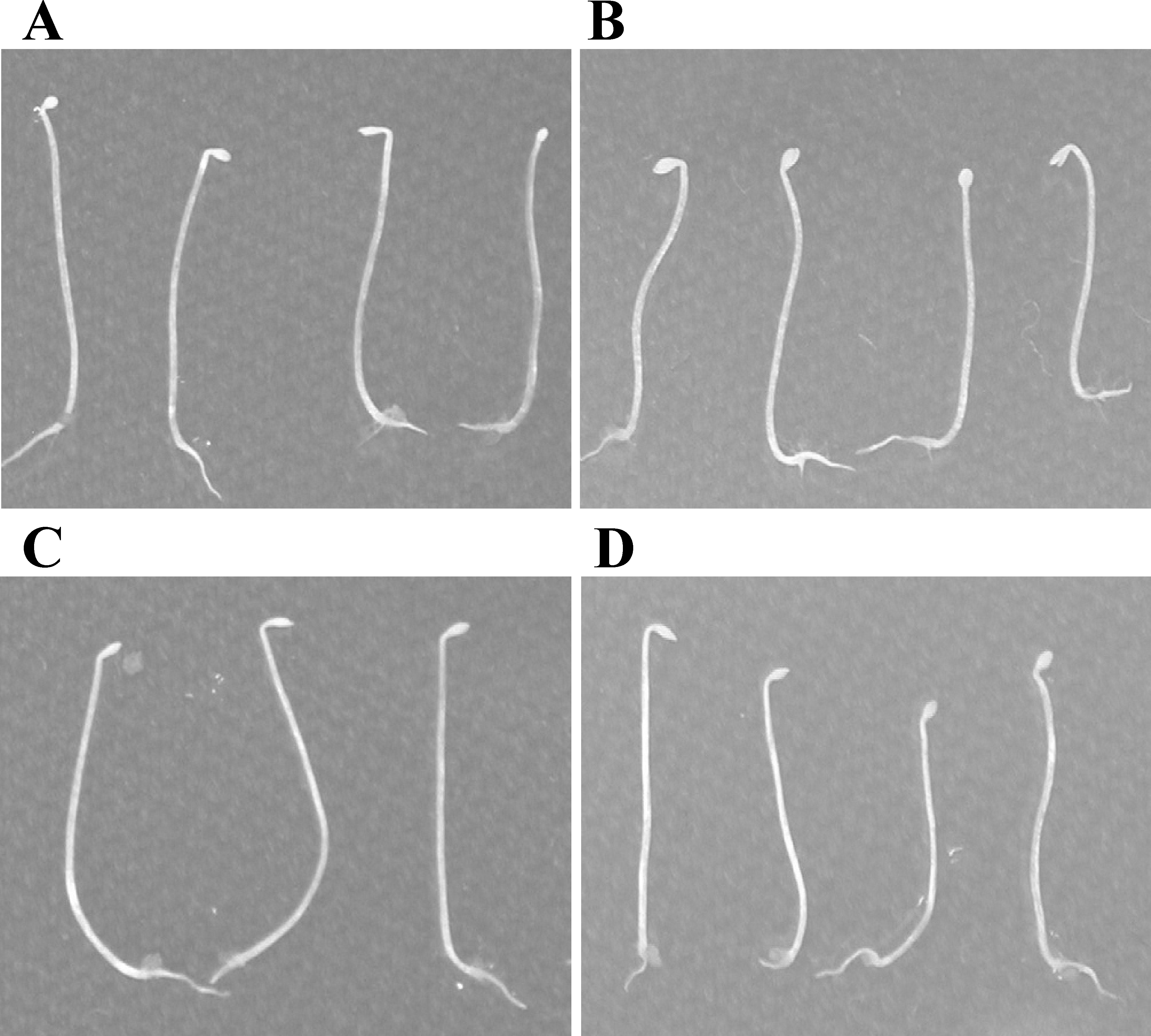

Supplement: Figure S2 — Overexpression of the PHYB-401:YFP fusion protein does not affect skotomorphogenesis of the transgenic seedlings. Arabidopsis seedlings were germinated according to the standard protocol (after 3 days stratification at 4°C seeds were imbibed and treated with 4 h white light) and then grown for 4 days in darkness. Phenotypes of seedlings expressing the PHYB:YFP (A) and PHYB-401:YFP (B) fusion proteins in phyB-9 background and those of the wild-type Col-0 (C) and phyB-401/phyA-201 (D) double mutant are shown. (TIF) [file pone.0027250.s002.tif]

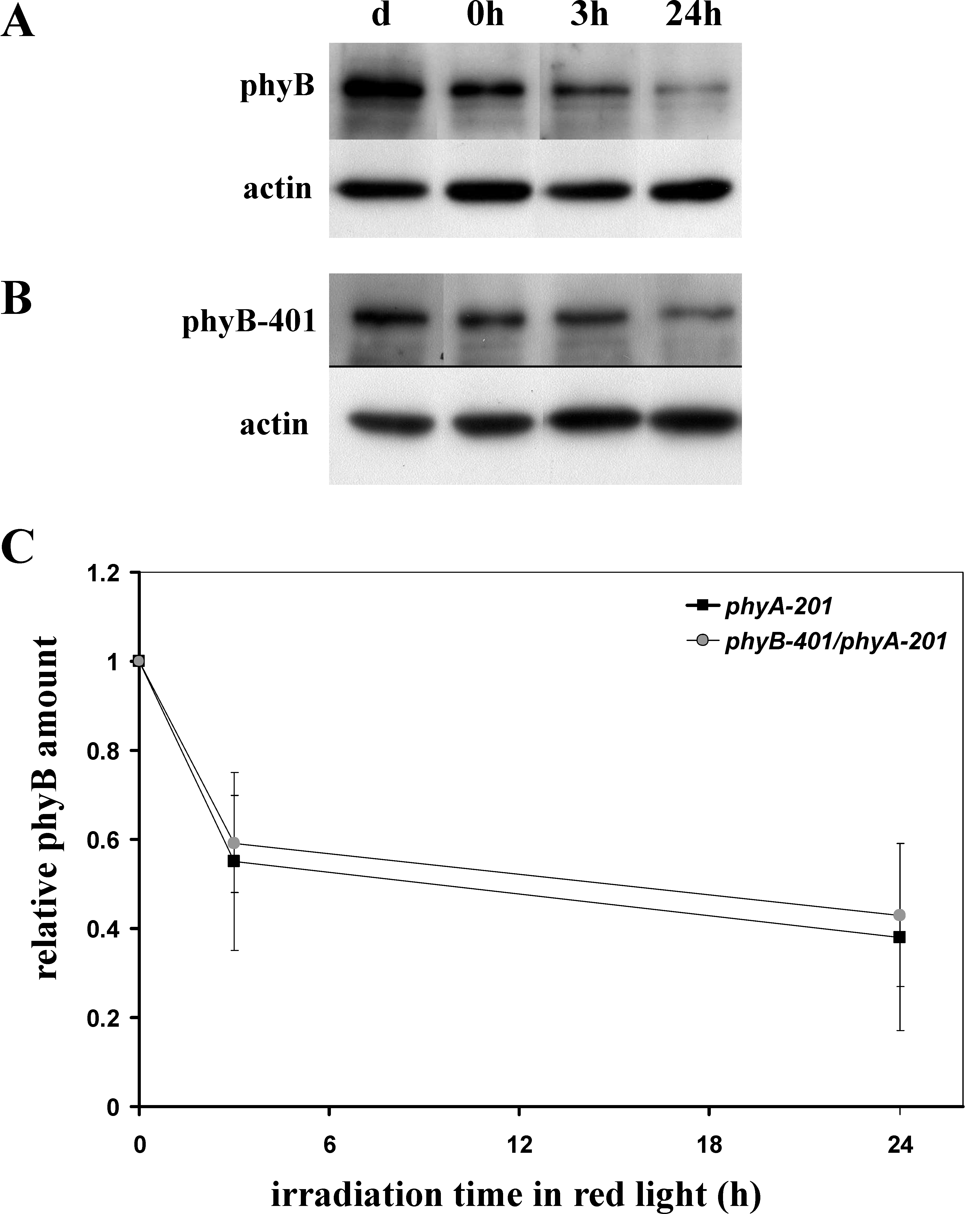

Supplement: Figure S3 — Degradation kinetics of the mutant and wild-type phyB do not differ significantly in cR. Accumulation levels of phyB and phyB-401 in phyA-201 background were analyzed by western blot hybridization using the monoclonal antibody B3B6 [41]. Degradation of the native phyB (A) and mutant phyB-401 protein (B) is shown in phyA-201 seedlings that were grown 4 days in darkness (d) and then irradiated with R light for 0, 3 and 24 h. The actin signals illustrate loading, the diagram (C) shows quantification of several western blots, error bars indicate standard error. (TIF) [file pone.0027250.s003.tif]

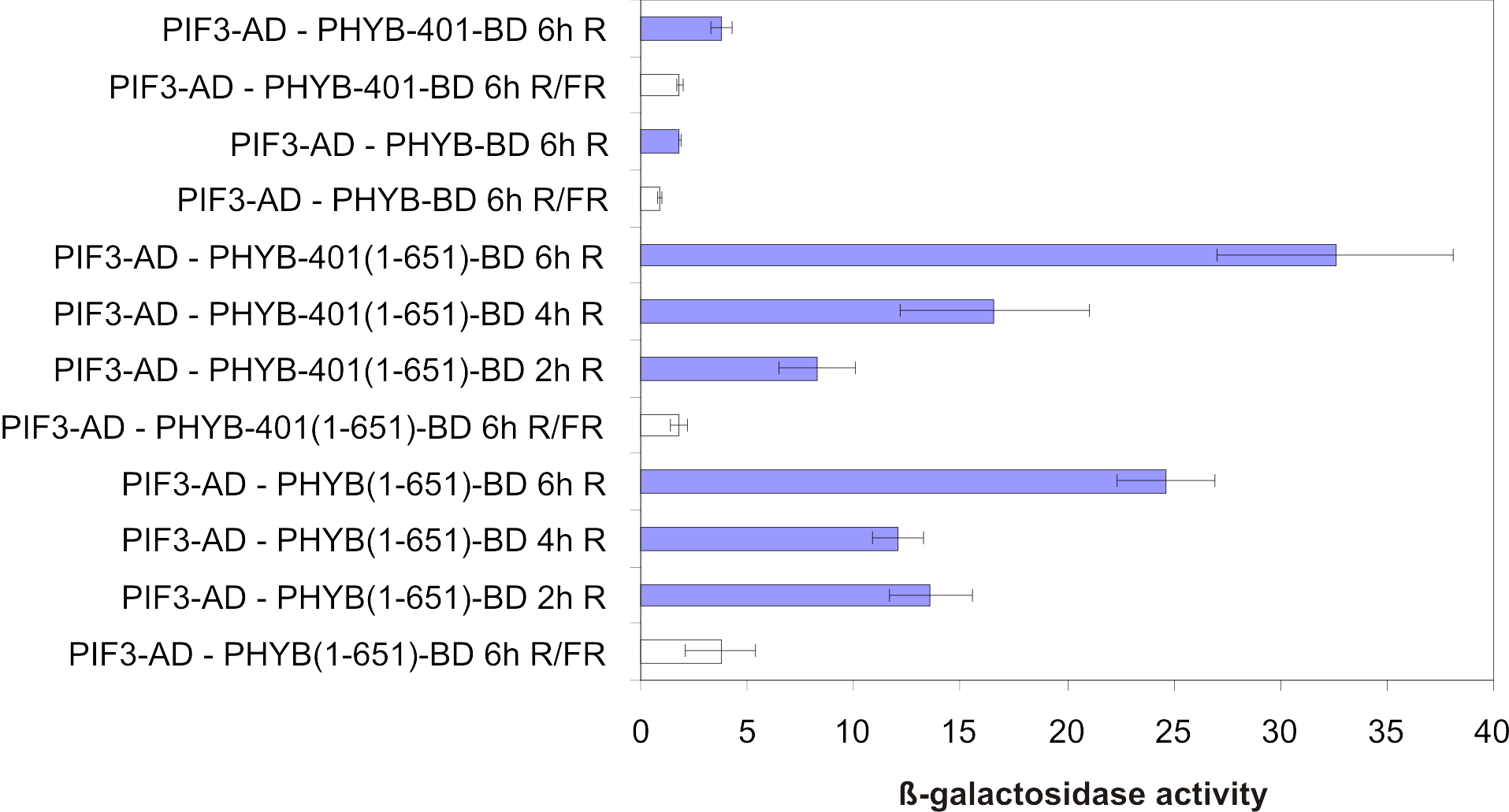

Supplement: Figure S4 — Interaction of PIF3 with phyB and with phyB-401 is identical. Liquid overnight cultures of yeast cells co-expressing the indicated proteins fused to the GAL4 activation (AD) or DNA-binding (BD) domain were treated with 5 min red light pulse (R) which was followed by a 5-min-long far-red light pulse (FR) in the indicated cases. After the given light pulses the cultures were incubated in the dark for the indicated time (between 2-6 h). Subsequently the ß-Galactosidase activity was determined. Error bars represent standard error of the mean. (TIF) [file pone.0027250.s004.tif]
